# Use of monomeric and oligomeric flavanols in the dietary management of patients with type 2 diabetes mellitus and microalbuminuria (FLAVA trial): study protocol for a randomized controlled trial

**DOI:** 10.1186/s13063-018-2762-9

**Published:** 2018-07-16

**Authors:** Mardin Rashid, Adrie J. M. Verhoeven, Monique T. Mulder, Reinier Timman, Yvonne van Beek-Nieuwland, Athumani A. Athumani, Adrienne A. M. Zandbergen, Hans E. van der Wiel, Eric J. G. Sijbrands, Kirsten A. Berk

**Affiliations:** 1 Department of Internal Medicine, Section of Pharmacology, Vascular and Metabolic Diseases, Erasmus Medical Center, PO Box 2040, 3000 CA Rotterdam, The Netherlands; 2 Department of Dietetics, Erasmus Medical Center, PO Box 2040, 3000 CA Rotterdam, The Netherlands; 3 Department of Psychiatry, Section of Medical Psychology and Psychotherapy, Erasmus Medical Center, PO Box 2040, 3000 CA Rotterdam, The Netherlands; 4 Department of Internal Medicine, Havenziekenhuis, Haringvliet 2, Rotterdam, 3011 TD The Netherlands; 5 General Practitioners Group, Stichting Zorg op Zuid, Maashaven Oostzijde 155, Rotterdam, 3072 HS The Netherlands; 6 Department of Internal Medicine, Ikazia Ziekenhuis, Montessoriweg 1, Rotterdam, 3083 HN The Netherlands; 7 Department of Internal Medicine, IJsselland Ziekenhuis, Prins Constantijnweg 2, Capelle aan de Ijssel, 2906 ZC The Netherlands

**Keywords:** Type 2 diabetes mellitus, Microalbuminuria, Monomeric flavanols, Oligomeric flavanols

## Abstract

**Background:**

Patients with type 2 diabetes mellitus (T2D) are prone to micro- and macro-vascular complications. Monomeric and oligomeric flavanols (MOF) isolated from grape seeds (*Vitis vinifera*) have been linked to improved endothelial function and vascular health. The aim of this study is to determine the effect of a daily supplementation of 200 mg MOF on renal endothelial function of patients with T2D and microalbuminuria.

**Methods/design:**

For this double-blind, placebo-controlled, randomized, multicenter trial 96 individuals (ages 40–85 years) with T2D and microalbuminuria will be recruited. Participants will be randomly assigned to the intervention group, receiving 200 mg of MOF daily for 3 months, or to the control group, receiving a placebo. The primary endpoint is the evolution over time in albumin excretion rate (AER) until 3 months of intervention as compared with placebo. Secondary endpoints are the evolution over time in established plasma markers of renal endothelial function—asymmetric dimethylarginine (ADMA), soluble vascular cell adhesion molecule-1 (sVCAM-1), soluble intercellular cell adhesion molecule-1 (sICAM-1), interleukin-6 (IL-6), and von Willebrand Factor (vWF)—until 3 months of intervention as compared with placebo. Mixed modeling will be applied for the statistical analysis of the data.

**Discussion:**

We hypothesize that T2D patients with microalbuminuria have a medically determined requirement for MOF and that fulfilling this requirement will result in a decrease in AER and related endothelial biomarkers. If confirmed, this may lead to new insights in the dietary management of patients with T2D.

**Trial registration:**

Nederlands Trial Register, NTR4669, registered on 7 July 2014.

**Electronic supplementary material:**

The online version of this article (10.1186/s13063-018-2762-9) contains supplementary material, which is available to authorized users.

## Background

Diabetes has become a worldwide epidemic. The estimated global prevalence of diabetes for adults between 20 and 79 years of age was 8.8% in 2017. This prevalence is expected to increase to 9.9% in 2045. About 4.0 million people between 20 and 79 years of age died from diabetes in 2017, resulting in a global all-cause mortality of 10.7% for people in this group [1, 2]. Patients with type 2 diabetes (T2D) are prone to develop micro- and macro-vascular complications [2, 3], reducing quality of life and putting a heavy burden on the health-care system [4]. At diagnosis, 6.5% of patients with T2D already show microalbuminuria; of these, 2.8% yearly progress into macroalbuminuria and 2.3% into nephropathy [5]. Microalbuminuria is a good proxy of endothelial dysfunction in patients with T2D and is strongly associated with an unfavorable cardiovascular and renal outcome [6]. Other established biomarkers for endothelial function are asymmetric dimethylarginine (ADMA), soluble vascular cell adhesion molecule 1 (sVCAM-1), interleukin-6 (IL-6), von Willebrand Factor (vWF), and soluble intercellular cell adhesion molecule 1 (sICAM-1), all of which have been related to renal endothelial health and vascular complications in T2D [7].

### Monomeric and oligomeric flavanols and their physiological effect

A recent publication of the ADVANCE (Action in Diabetes and Vascular Disease: Preterax and Diamicron Modified-Release Controlled Evaluation) trial showed that moderate consumption of alcohol, particularly wine, was associated with reduced cardiovascular events, microvascular complications, and all-cause mortality in patients with T2D [8]. Over the past decades, researchers have wondered which compounds of wine could be responsible for the lower risk of cardiovascular disease. Lately, the notion that the presence of monomeric and oligomeric flavanols (MOF) could explain the cardio-protective effects of red wine has found broad acceptance [9]. In the human diet, a broad variety of single and condensed forms of MOF, ranging from monomers (catechins) and oligomers (oligomeric proanthocyanidins, or OPCs) to polymers, is found in red wine, tea, cacao, legumes, and bark, peels, and skins of many plants [10]. In a report in 2012, the European Food Safety Authority (EFSA) acknowledged that cacao flavanols maintain endothelium-dependent vasodilation [11]. However, the MOF level in the average Western diet tends to be low [12].

In a prospective cohort study (European Prospective Investigation into Cancer and Nutrition, or EPIC), a significant inverse trend was observed between MOF intake and risk of T2D [13]. Another trial showed a significant association between the intake of polyphenols, of which MOF are a subclass, and a decreased risk of cardiovascular disease (CVD) [14]. Higher intakes of anthocyanins (red pigments of which condensed flavanols are the precursors) and flavones (a class of flavonoids) have been associated with significant improvements in insulin sensitivity and high-sensitivity C-reactive protein (hs-CRP) levels [15]. Grape seed extracts containing relevant concentrations of MOF significantly decreased systolic blood pressure and CRP level in patients with T2D [16]. A daily supplementation of 150 mg of MOF significantly reduced several microvascular abnormalities in patients with diabetic nephropathy and retinopathy [17, 18]. In a recent study, use of 200 mg MOF per day for 8 weeks resulted in vascular health benefits in “healthy” male smokers [19]. In the same study, MOF modulated the expression of genes associated with cardiovascular disease pathways, mainly genes involved in chemotaxis, cell adhesion, cell infiltration, and cytoskeleton organization, suggesting diminished immune cell adhesion to endothelial cells [20]. Whether MOF have a physiological effect on the renal endothelial health of patients with T2D has not yet been established.

## Objective

The objective of this study is to assess the effects of daily supplementation of 200 mg MOF on renal endothelial function in T2D patients with microalbuminuria.

## Methods/design

### Study design

**Fig. 1:**
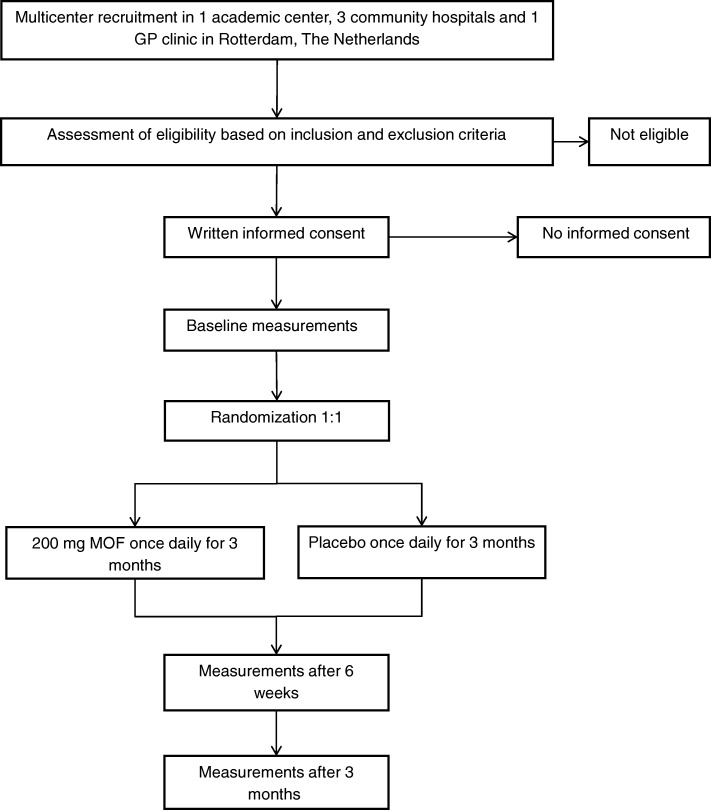
Flow chart of the study

This study is a double-blind, randomized, controlled, multicenter trial. After giving written informed consent, eligible participants will be randomly assigned to placebo or intervention for 3 months. Outcome parameters will be measured at baseline, at 6 weeks, and at 3 months. In Fig. 1, we show the flow chart of this study. The SPIRIT (Standard Protocol Items: Recommendations for Interventional Trials) checklist for study protocols is provided as an Additional file 1.

### Study population

Patients with T2D will be recruited from the diabetes outpatient clinic of the Erasmus Medical Center in Rotterdam and the community hospitals of Havenziekenhuis, IJsselland Ziekenhuis and Ikazia Ziekenhuis as well as GP-clinic Stichting Gezond op Zuid in Rotterdam. Patients who attend the diabetes outpatient clinics of the participating centers will be screened for eligibility through assessing their medical records by the study coordinator (MR). Then eligible patients are informed by their designated physician about the trial during a routine control appointment. If the patients are interested, the study coordinator provides them with a flyer and additional information at the outpatient clinic. Hereafter, patients will be given at least 2 weeks to decide whether they want to participate. Inclusion is complete after signing the informed consent form.

### Definition of T2D

Information on the diagnosis of T2D was obtained from patient’s medical records. Diabetes was defined according to the World Health Organization [21] and the American Diabetes Association [22] guidelines as at least one of the following:

- Fasting plasma glucose of at least 7.0 mmol/L, measured at at least two separate time points
- Non-fasting plasma glucose level of at least 11.1 mmol/L, measured at at least two separate time points
- HbA_1c_ of at least 48 mmol/mol, measured at at least two separate time points
- Using anti-diabetic medication
- Diagnosis of T2D documented in the patient’s medical record by the designated physician.

The information was checked by the study coordinator. In case of uncertainty, the patient’s treating physician was consulted.

### Inclusion criteria

- T2D, as defined in the section above
- Age of 40–85 years
- Microalbuminuria in the previous 6 months (as microalbuminuria can change during time, results should not be older than 6 months), defined as one of the following:
- 30–300 mg albumin in a 24-h urine sample
- or 3.5–35 mg albumin/mmol creatinine in females and 2.5–25 mg albumin/mmol creatinine in males in a urine portion.

This definition is derived from the Dutch national guidelines [23].

### Exclusion criteria

- Other types of diabetes mellitus as derived from the medical records
- Prior (less than 4 weeks before participating) or current use of any specific dietary supplementary products providing daily amounts of MOF of 25 mg/day or higher
- Anticoagulation medication
- Major health conditions: organ transplantation, untreated cancer, current chemotherapy or radiotherapy, or acute or chronic organ failure
- Microalbuminuria due to conditions other than T2D
- Pregnancy or lactation during the trial.

### Randomization and blinding

Patients will be randomly assigned to either intervention or control group at an allocation ratio of 1:1. All investigators, medical staff, statisticians, and participants are blinded to the intervention. The statistician generates the allocation sequence and delivers it to the nutritional company responsible for the preparation of the product and placebo. The nutritional company produces the research product and placebo using the same packages without labels of the company or product name. We do not mention the product or company’s name to the participants. The sachets are packaged in boxes containing the appropriate amount for a 3-month intervention of an individual subject. The boxes are labeled with a unique numerical code, derived from the generated allocation sequence. They will be given to the participants in consecutive order. To avoid bias, the consecutive order of the labeled boxes is strictly followed. Thus, the first recruited patient is handed box number 1, the second patient is handed box number 2, and so on, regardless of the center of recruitment. Furthermore, the nutritional company prepares two identical sealed boxes, each containing 114 numerical coded envelopes with the respective treatment allocation inside. One sealed box will be stored at a secured place where only the principal investigator has direct access; the other sealed box will be kept at a secured place at the office of the head of the department. Only in case of a medical urgency is the blinding code to be broken. The designated physician and the principal investigator are ultimately responsible for the final decision of breaking the code for a single subject. Otherwise, decoding will first take place after a blind review including preliminary statistical analyses of the primary outcome data by an independent statistician. In all cases, unblinding will take place in the presence of a witness.

### Sample size calculation

Considering a clinically relevant effect of 20% change of albumin excretion rate (AER), which is half the effect of angiotensin-converting enzyme inhibitors (ACEis) on AER [24, 25], we estimated that the average AER of 100 decreased to 80 μg/min albumin (standard deviation 40) in the intervention group compared with no expected change in the placebo group. With a two-sided alpha set at 0.05 and power at 0.80 and a correlation of 0.70 between the three repeated measures, we calculated that at least 48 patients in each study group (i.e. 96 in total) would be required [26].

#### Intervention and control

##### Research product, treatment, and placebo

Masquelier’s^®^ Endoclair^®^ (abbreviated as MOF) was designed as Food for Special Medical Purpose by I.N.C. Agency B.V. (Loosdrecht, The Netherlands) in accordance with EU Commission Directive 1999/21/EC of 25 March 1999 on dietary foods for special medical purposes. Its active ingredient is 200 mg of catechins and epi-catechins (single flavan-3-ols) and oligomeric flavan-3-ol units (dimers to pentamers) per sachet. The MOF have been extracted from *Vitis vinifera* (grape seeds). Detailed analyses have established that the preparation is standardized to contain about 85% (wt/wt) flavan-3-ols (as established with the vanillin-H_2_SO_4_ assay [27]), of which 50% to 60% (wt/wt) are single and dimeric flavan-3-ols (determined by high-performance liquid chromatography), and polymeric proanthocyanidins (hexamers and beyond) are below detection limit. The manufacturer guarantees the stability of the product composition until the expiration date. For the manufacturing of the placebo, only the active ingredient (MOF) is removed from the product. Product and placebo will be from the same batch. The placebo product has the same package, taste, and color as the research product (Table 1). When the white powder is dissolved in water, a similar yellowish solution is obtained with both preparations.

**Table 1:** Ingredients of the placebo and the research product

Formulation placebo	
Sweetener (isomalt GS-PF; E953)	1400 mg
Food acid (citric acid; E330)	200 mg
Black tea extract	150 mg
Natural lemon flavor	20 mg
Anti-caking agent (tricalcium phosphate) E341(iii))	10 mg
Acidity regulator (tripotassium citrate))) E332(ii))	10 mg
Sweetener (sucralose; E955)	10 mg
Total	1800 mg
Formulation Endoclair^®^	
Sweetener (isomalt GS-PF; E953)	1400 mg
Monomeric and oligomeric flavanols	200 mg
Food acid (citric acid; E330)	200 mg
Black tea extract	150 mg
Natural lemon flavor	20 mg
Anti-caking agent (tricalcium phosphate) E341(iii))	10 mg
Acidity regulator (tripotassium citrate) E332(ii))	10 mg
Sweetener (sucralose; E955)	10 mg
Total	2000 mg

In the composition of both the investigational product and the placebo, 150 mg of black tea extract (BTE) is included for taste and color reasons only (Table 1). The amount of the total polyphenols in the BTE is about 30 mg, the same amount as in one cup of tea. This amount is the same for the placebo and the investigational product and thus does not influence our results.

In its quality control process, the manufacturer uses “Complex Phytonutrient Authentication” via nuclear magnetic resonance and principal component analysis to authenticate the quality and standardized amount of the MOF fractions. For the exact content and nutritional value per sachet, see Tables 1 and 2.

**Table 2:** Nutritional value per sachet of placebo and research product

Energy	4.12 kcal
Fat	0.0 g
Carbohydrates	1.5 g (polyols)
Protein	0.0 g
Sodium	0.0 mg

The dose of MOF and the duration of the intervention in the current trial were based on data from previous prospective human studies, in which MOF amounts between 100 and 300 mg per day for durations from 15 to 90 days were used. In these studies, positive effects on cardiovascular health without serious side effects were observed [19, 20]. Therefore, a dosage of 200 mg and a treatment duration of 3 months in the current study are most likely sufficient in order to address the objective. There is a hypothetical risk for patients using anticoagulation medications: the product could have a mild and dose-dependent platelet-inhibiting effect [28]. Therefore, to avoid possible interactions, we decided to exclude all patients using anticoagulants. As side effects, nausea and epigastric discomfort when starting with the product have been reported in a minority of patients. These side effects are most likely caused by the used sweeteners and therefore will be the same for both the control and intervention groups. Side effects will be monitored during the trial.

We instruct the patient to take the daily dosage at the same time point each day, once a day, preferably half an hour before a meal or in between meals. The product should be dissolved in a glass of tap water at room temperature and be drunk immediately. In case of gastrointestinal discomfort while using the product, splitting the dosage might help lessen the discomfort. In that case, the patient may split the daily dosage into two dosages. During the 3-month study period, we will regularly contact participants by phone to encourage them to complete the study. This will also be done face-to-face at the three measurement moments. Compliance will be assessed by questionnaires in which participants are asked for the number of sachets they forgot to use and for which reason.

##### Study procedure

**Fig. 2:**
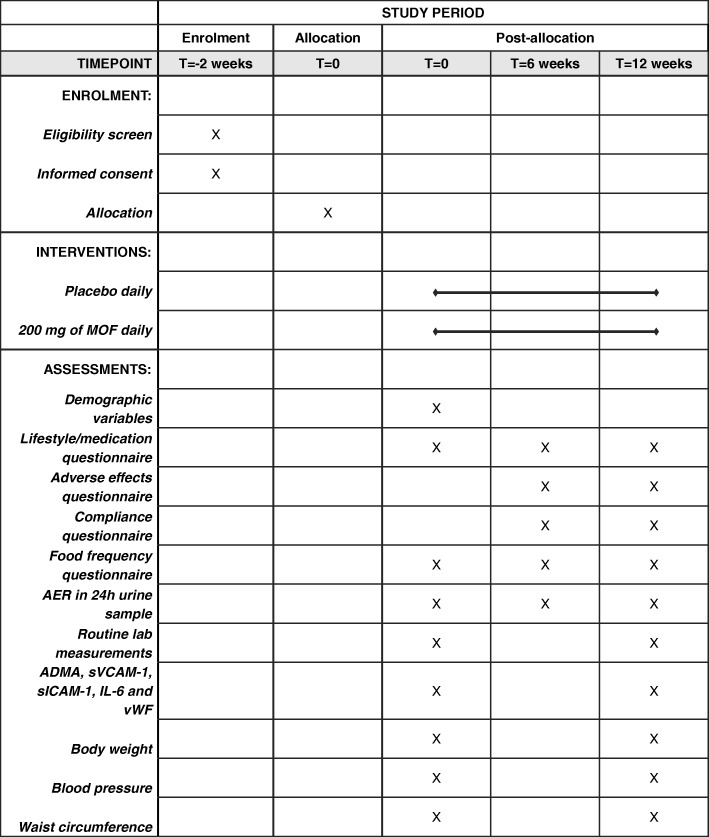
Schedule of enrolment, interventions, and assessments. Abbreviation: *SPIRIT* Standard Protocol Items: Recommendations for Interventional Trials

At baseline, 6 weeks, and 3 months, 24-h urine will be collected for AER measurements. Information about demographic variables, lifestyle, adverse effects, compliance, and food intake will be collected by using standard questionnaires. The urine collection procedure starts in the morning and lasts 24 h; it stops at the same time point it started the day before. During collection, all of the produced urine is collected in a container that was handed out to the patient at the outpatient clinic. Within a couple of hours after the collection, urine is delivered in the outpatient clinic. AER is measured the same day as soon as possible after delivery. The patient is instructed to keep the container with urine in a dark and cool place at room temperature until delivery. At baseline and after 3 months, blood samples will be collected for determination of ADMA, sVCAM-1, sICAM-1, IL-6, and vWF and for measuring HbA_1c_ fasting glucose, lipids, creatinine, and estimated glomerular filtration rate (eGFR). Non-fasted venous blood is collected in EDTA tubes at a random moment of the day at the outpatient clinic. Plasma samples will be stored at −80 °C until analysis. Although patients are not required to fast before blood collection, we do keep a record of whether they fasted or not. In addition, body weight, blood pressure, and waist/hip circumference will be measured. In Fig. 2, we show a schedule of the study procedures. All participants receive usual care as provided by their diabetes team and must continue all of their prescribed medication, including adjustments when required. Since this may potentially affect our outcome measures, all used medications, dosage modifications, and other medical interventions during the trial will be recorded to adjust for this potentially confounding effect. We will ask the participants to keep their current lifestyle unchanged. There are no dietary restrictions during the study, except for the use of specific dietary supplement products containing at least 25 mg MOF. To control for dietary flavanols in regular food, we will use detailed dietary questionnaires to calculate the amounts.

#### Outcome measures

##### Primary outcome measure

The primary endpoint is difference in evolution of AER from baseline to 3 months of intervention in absolute values as compared with placebo. AER will be measured in a 24-h urine sample at the clinical chemistry laboratory of the Erasmus Medical Center. The albumin level in urine will be measured by immunoturbidimetric assay (Roche Diagnostics GmbH, Mannheim, Germany).

##### Secondary outcome measures

The difference between intervention and placebo in change of AER expressed as percentage of baseline AER will be taken as a secondary outcome. Other secondary endpoints are the differences in the course of the established plasma markers of renal endothelial function (ADMA, sVCAM-1, sICAM-1, IL-6, and vWF) from baseline to 3 months. These markers will be measured by using enzyme-linked immunosorbent assays.

##### Other study parameters

HbA_1c_, fasting glucose, lipids, eGFR, and plasma creatinine will be measured at the clinical chemistry laboratory of the Erasmus Medical Center by using routine lab techniques.

##### Data monitoring

All data will be filed and managed by using the trial management system OpenClinica (Waltham, MA, USA). Data will be entered into OpenClinica with a consecutive code number (no initials or birth date). Human material (blood samples) will also be encoded and kept at −80 °C until analysis. The subject identification code list, which links the code number to the participant, will be safeguarded by the secretary of the department of internal medicine in a “master file”. The key to the code is accessible by the investigators only. Study data can be accessed only by the investigator team, staff of the Health Care Inspection, and members of the Medical Ethical Committee, as stated in the informed consent form. The handling of personal data will comply with the Dutch Personal Data Protection Act (in Dutch: De Wet Bescherming Persoonsgegevens, WBP). All data, urine, and blood samples will be kept for a maximum of 15 years after the trial has finished.

An independent data monitoring committee (DMC) will be established in accordance with research integrity guidelines of the Erasmus Medical Center. The current trial has been identified as a trial with minimal risk for the participants and therefore the frequency of monitoring by the DMC will be only once a year. During this monitoring visit, the following items will be checked: the trial master file, inclusion speed, informed consent (sample of 10% of the participants), inclusion and exclusion criteria (sample of 1–10%), compliance with the protocol (sample of 1–10%), source document verification (sample of 1–10%), (serious) adverse events (sample of 1–10%), instructions of study procedures, certification of laboratories involved, and the labeling and storage of biological samples.

##### Public disclosure and publication policy

The results of this trial will be disclosed unreservedly. We registered the trial before its start in the Netherlands Trial Registry. Our intention is to publish the results as soon as possible after completion of the sample analyses and data evaluation in an appropriate peer-reviewed scientific journal.

##### Safety

In case of a suspected serious adverse event (SAE), the investigator will report the SAE to the sponsor without undue delay after obtaining knowledge of the event. Via the web portal ToetsingOnline, the sponsor will report the SAEs to the accredited METC (the medical research ethics committee, which approved the protocol) within 7 days of first knowledge for SAEs that result in death or are life-threatening followed by a period of a maximum of 8 days to complete the initial preliminary report. All other SAEs will be reported within a period of 15 days after the sponsor has first knowledge of the SAEs. Because of the low risk associated with the investigational product, no data and safety monitoring board or safety committee will be established.

##### Statistical analysis

All analyses will be conducted in accordance with the intention-to-treat principle. We will use the mean (in case of normal distribution) and median values (in case of non-normal distribution) as measures of central tendency for numerical data and the standard deviation and interquartile range as measures of variability, respectively. Mixed modeling will be applied for longitudinal analyses of the data. Mixed modeling can efficiently handle data with missing and unbalanced time points. Three levels in the models will be postulated. The participating centers constitute the upper level, the patients the middle level, and their repeated measures the lower level. First, for each outcome variable, a model will be postulated, and the primary or secondary outcomes will be dependent variables. The models will include treatment group, time, and logarithm of time and treatment-time interactions as fixed effects. The deviance statistic [29] using restricted maximum likelihood [30] will be applied to determine whether the covariance structure should also include slope and intercept-slope interaction next to the intercept. No interim analysis will be performed. Analyses will be carried out by using SPSS version 21.0 (IBM, Armonk, NY, USA) (http://www-01.ibm.com/support/docview.wss?uid=swg21608060).

## Discussion

The presence of microalbuminuria is associated with endothelial dysfunction and poor cardiovascular outcome in T2D [31]. Although microalbuminuria is associated with a number of other risk factors, it is considered an independent risk factor for CVD and cardiovascular events in diabetes [31]. The exact mechanism leading from microalbuminuria to end organ damage is not yet completely understood. Endothelial dysfunction, however, might play a crucial role in this process [32]. Hypothetically, microalbuminuria is a signal of endothelial dysfunction leading to increased permeability of atherogenic lipoprotein particles [31]. In addition to endothelial dysfunction, a number of metabolic disorders are associated with microalbuminuria leading to cardiovascular damage: transvascular leaking of albumin in vessels, elevated glomerular filtration rate, elevated blood pressure, hyperinsulinemia, elevated plasma fibrinogen levels, and altered blood lipid levels and function may also play important roles [31, 32].

Treating microalbuminuria by improving endothelial function is most probably beneficial in patients with T2D. Although ACEis and angiotensin receptor inhibitors (angiotensin receptor blockers, or ARBs) are effective treatments to achieve this goal [31, 32], use of MOF as part of the dietary management may also be an effective auxiliary strategy. The individual responses to ACEis and ARBs show large variation. They are not equally effective in treating microalbuminuria in each individual patient [32]. Besides, a remarkable percentage of treated patients with T2D will eventually develop diabetic kidney disease during the course of the disease [33]. Therefore, if MOF are proven to fulfill the specific nutrient requirement, they may become a dietary tool to manage albumin excretion in patients with T2D and microalbuminuria in general and in the insufficient responders to ACEis and ARBs in particular.

Obviously, the safety features of MOF are very important. In contrast to ACEis and ARBs, Endoclair MOF is registered as a food supplement for special medical purposes, not requiring patient monitoring upon first application. The advantageous effects of MOF on reversing endothelial dysfunction and maintaining cardiovascular health have been widely addressed in past decades; in epidemiological studies, average dietary intake of flavanols has been linked to health status [9, 13–16, 19, 20, 32, 34]. Nevertheless, there is a lack of clinical and well-conducted studies specifically addressing the effects of MOF on renal endothelial function in T2D.

We hypothesize that the use of 200 mg MOF daily for 3 months will have a beneficial effect on renal endothelial function and will result in a decrease of AER. This may lead to new insights into the dietary management of T2D patients with renal complications. MOF could have beneficial effects on endothelial function in other parts of the body as well, as suggested by previous studies [9, 13–16, 19, 20, 32, 34]. Finally, since the participants in this trial are recruited from tertiary, secondary, and primary referral centers, our study sample well represents T2D patients with microalbuminuria in general.

## Trial status

Protocol version number 3.1, June 5, 2018.

Start recruitment: January 1, 2015.

End of recruitment (estimated): December 31, 2018.

Trial sponsor: Erasmus Medical Center, Rotterdam, The Netherlands.

## Additional file

Additional file 1: SPIRIT 2013 Checklist. (DOC 121 kb)

## Abbreviations

ACEi: Angiotensin-converting enzyme inhibitor
ADMA: Asymmetric dimethylarginine
AER: Albumin excretion rate
ARB: Angiotensin receptor blocker
BTE: Black tea extract
CVD: Cardiovascular disease
DMC: Data monitoring committee
eGFR: Estimated glomerular filtration rate
IL-6: Interleukin-6
MOF: Monomeric and oligomeric flavanols
SAE: Serious adverse event
sICAM-1: Soluble intercellular cell adhesion molecule-1
sVCAM-1: Soluble vascular cell adhesion molecule-1
T2D: Type 2 diabetes mellitus
vWF: Von Willebrand Factor
